# The Integration of Information Technology in the Management and Organization of Nursing Care in a Hospital Environment: A Scoping Review

**DOI:** 10.3390/ijerph21080968

**Published:** 2024-07-24

**Authors:** Dulce Cachata, Mónica Costa, Teresa Magalhães, Filomena Gaspar

**Affiliations:** 1 Innovation Health Center, Hospital de Cascais, 2755-009 Alcabideche, Portugal; 2 Nursing Research, Innovation and Development Center in Lisbon (CIDNUR), Nursing School of Lisbon, 1990-096 Lisbon, Portugal; mcrcosta81@gmail.com (M.C.); mfgaspar@esel.pt (F.G.); 3 Escola Superior de Saúde do Instituto Politécnico de Setúbal, 2914-503 Setúbal, Portugal; 4 NOVA National School of Public Health, Public Health Research Centre, Comprehensive Health Research Center (CHRC), NOVA University of Lisbon, 1600-560 Lisbon, Portugal; teresa.magalhaes@ensp.unl.pt

**Keywords:** nurses, information technology, management and organization, nursing care

## Abstract

The adoption of health technologies is occurring on an unprecedented scale, with enormous potential to improve the health of populations. In this context, information technology (IT) in nursing has emerged with a focus on quality and safety of care for the benefit of the patient. There is insufficient reliable evidence demonstrating how the integration of IT in nursing care influences methodologies for managing and organizing care in terms of structure and care practice, justifying a scoping review that synthesizes the knowledge produced so far. Online databases were used to identify papers published in 2012–2023, from which we selected nine publications that used information technology in the nursing care processes. The participants were hospital nurses and nurse managers. The results show that the integration of IT in healthcare organizations impacts the management and organization of nursing care, and changes in structure, process reorganization, management, training, and the development of nurses’ skills. To minimize this impact, the organizational structure must be prepared for a cultural change, with well-defined and communicated policies and procedures, and strong leadership. Within the teams, the importance of process reorganization, continuous training, and skill development emerges, thus enhancing the integration of IT into practice environments in conjunction with care.

## 1. Introduction

Florence Nightingale was a milestone figure in the history of nursing and a pioneer in her concern for records related to the quality of care provided by nurses. She used these records to identify and promote improvements in deficient areas, highlighting the importance of documenting information about patients, disease status, care provided, or patient’s evolution. In this way, she emphasized the uniqueness of nurses’ notes [1]. In the 1970s, decision support systems began to be incorporated into nursing practice. Since then, these systems have become crucial intellectual tools for nurses, as they have enabled them to obtain individualized information, supporting them and enhancing the quality of care [2]. In 2001, Brazeal stated that people are often afraid that technology will make humans less human [3]. This statement resonates as a major concern in nursing, particularly when it comes to understanding the individuals they care for [4,5]. The concept of technology as something natural that, when enhanced, increases efficiency in care is fundamental to nursing practice [3]. Information, as a source of knowledge, currently represents a valuable resource within the organization and should be seen as a product that must be defined, measured, analyzed, and improved [6]. Simultaneously, it should be based on quality and safety standards, enhancing care processes in the hospital [7,8]. The roles and responsibilities of management in healthcare organizations regarding technological integration are often mentioned in the literature [9], especially with regard to nursing care, reinforcing the importance of understanding how nurses manage care organization processes [10], based on a theoretical–methodological framework [11], and identifying elements associated with person-centered care and its quality [12]. 

The plurality of knowledge in nursing involves reaffirming locally produced knowledge, so-called local knowledge, knowledge produced in context. Some studies show that nurses working in hyper-technological environments value high-touch environments more than high-tech environments, recognizing that patients need human interaction, engagement in care, acknowledgment of their own value, and the ability to participate in therapeutic decisions [6]. The idea of person-centered care needs to be reconfigured in the relational spaces of technology incorporated into nursing clinical practice. It is not just about transforming the use of health technologies into person-centered practice. The dynamic triad of the nurse–patient–technology relationship in an interpersonal space must be considered as a whole [6]. The implementation of Nursing Information Systems has generated the need for nurses to develop new skills in care delivery [8], increasingly requiring nurses to be involved in the strategic planning processes for their implementation [13]. The importance of data produced and documented because of nurses’ professional practice has posed a huge challenge to the development of Nursing Information Systems in recent decades, essentially due to the increasing need for information and the difficulties in managing it [14]. A review of the impact of technology on the management and organization of nursing care in a hospital environment, aiming for excellence in nursing care, emerges as important.

This scoping review aimed to identify and map, in the scientific evidence, nurses’ use of information technology and its impact in the management and organization of nursing care in a hospital environment. The following research question was formulated: How does the use of information technology impact the management and organization of nursing care in a hospital environment?

## 2. Materials and Methods

A scoping review was used, incorporating quantitative and qualitative studies available to address review questions. This method allows a review to extract different data and develop them in a meaningful, transparent, and systematic way [15]. The reports followed PRISMA ScR (Preferred Reporting Items for Systematic Reviews and Meta-Analyses extended for Scoping Reviews) guidelines to structure the collected information [16].

### 2.1. Protocol and Registration

Before this review, a protocol was drafted and registered in the Open Science Framework (https://osf.io/tqvyd/, accessed on 3 March 2023) and a preliminary search was carried out in Prospero, JBI EBP, and the Cochrane Library to confirm the absence of an identical protocol. 

### 2.2. Information Sources

The research was carried out in three distinct phases using the Medline, CINALH, and Cochrane databases via the EBSCO platform, ScienceDirect, Wiley Online Library, Scielo, and PubMed. Data extraction and subsequent analysis were performed using the JBI Template Source of Evidence Details, Characteristics and Results Extraction Instrument, adapted to the question of this review: How does technology impact the management and organization of nursing care in a hospital environment?

For this review, two research sub-questions were posed:

- How do healthcare institutions integrate technology into their organizational structure and processes?
- How do nurses use technology to provide care?

### 2.3. Search

Firstly, indexing terms were identified in the CINAHL and MEDLINE databases to find the most suitable words and terms to construct the Boolean phrase.

The second phase of the research was a conventional search in electronic databases using the previously defined terms. The databases used were Medline, CINALH, Cochrane Central Register of Controlled Trials through the EBSCO platform, ScienceDirect, Wiley Online Library, Scielo, and PubMed. The search strategy was conducted using different combinations of descriptors, employing the Boolean operators “AND” and “OR”, and the “*” tool to truncate words and contain multiple suffixes, with publication dates from January 2012 to May 2023 set as the limiting filter (Table 1). To access documents not included in the databases identified above, a search was also carried out in the so-called gray literature, aiming to include information and studies that met the predefined inclusion criteria but were not included in scientific publications. All types of studies published in Portuguese, French, Italian, English, and Spanish (Castilian) were considered eligible.

### 2.4. Selection of Sources of Evidence

In the third stage, the reference of selected articles was analyzed, reading studies considered relevant and, consequently, additional, to increase the sensitivity of the research and reach the broadest possible spectrum of the literature produced. No restrictions were imposed on the type of studies to be analyzed, and all studies considered relevant to the topic at hand were included in the review, regardless of the research design adopted. 

Inclusion criteria: We included all types of studies demonstrating the integration of technology in nursing care and its relationship with organizational structure and processes, as well as the management and organization of care in a hospital environment.

### 2.5. Eligibility Criteria

The inclusion criteria used, explained below, were constructed based on the PCC mnemonic—Population, Concept, and Context—which refers to the type of study, language, and publication date of the documents.

Population Nurse/Nurse Manager: They have the scientific, technical, and human competence to provide nursing care to individuals, families, groups, and communities, at the levels of primary, secondary, and tertiary prevention [17], throughout the life cycle. A nurse’s competence refers to the professional performance that demonstrates the effective use of knowledge and skills that enable clinical judgment and decision making, and in the areas of management, research, teaching, training, and consultancy, it contributes to the improvement and evolution of nursing care delivery [17]. 

ConceptInformation Technology: Health technology is the application of knowledge and skills organized in the form of devices, procedures, and systems developed to solve a health problem and improve the quality of life [18]. Information technologies in nursing are essentially used to support the nursing process [19]. They make data available, ensuring that this data flow maintains the meaning they convey, using common languages and protocols [14], with an information processing model: input, processing, and output of data [20]. Guided by technical and semantic interoperability standards [21], these technologies strategically manage information within the team. They enable alternatives to traditional paper-based systems, ensuring organized and accessible information. This facilitates communication, promotes efficiency and productivity, supports effective care, and assists decision making [8]. 

ContextNursing care: This includes autonomous or interdependent interventions to be performed by nurses within the scope of their professional qualifications. In the practice of care, nurses need to focus their intervention on the complex interdependence of person and environment [22]. The professional practice of nursing focuses on the interpersonal relationship between a nurse and a person or a nurse and a group of people. Nursing care has its focal point on promoting the health projects that each person lives and pursues, bearing in mind that good care means different things to different people. In the management and organization of nursing care, nurses organize, coordinate, execute, supervise, and evaluate nursing interventions, decide on techniques and means to be used in care delivery, leveraging and making the most of existing resources [6]. 

This study will, therefore, consider nurses and nurse managers in hospital environments who integrate information technology into their care practices.

### 2.6. Data Charting Process

Two independent reviewers conducted the selection of studies, data extraction, and coding. Covidence^®^ software (https://www.covidence.org/, (accessed on 14 July 2024)) was used to manage the search results, enabling seamless collaboration in the study selection process. Duplicate studies were automatically removed. Initially, the titles and abstracts of the selected studies were analyzed according to the inclusion criteria described above. After the first selection, the articles were read in full. Where both reviewers disagreed, each justified their choice, and consensus was reached. 

### 2.7. Synthesis of Results

Finally, after both reviewers had read the eligible studies in full, the relevant data were extracted and subsequently analyzed using the JBI Template Source of Evidence Details, Characteristics and Outcomes Extraction Tool, adapted to the question of this review, validated, and accepted by both reviewers. 

## 3. Results

**Figure 1:**
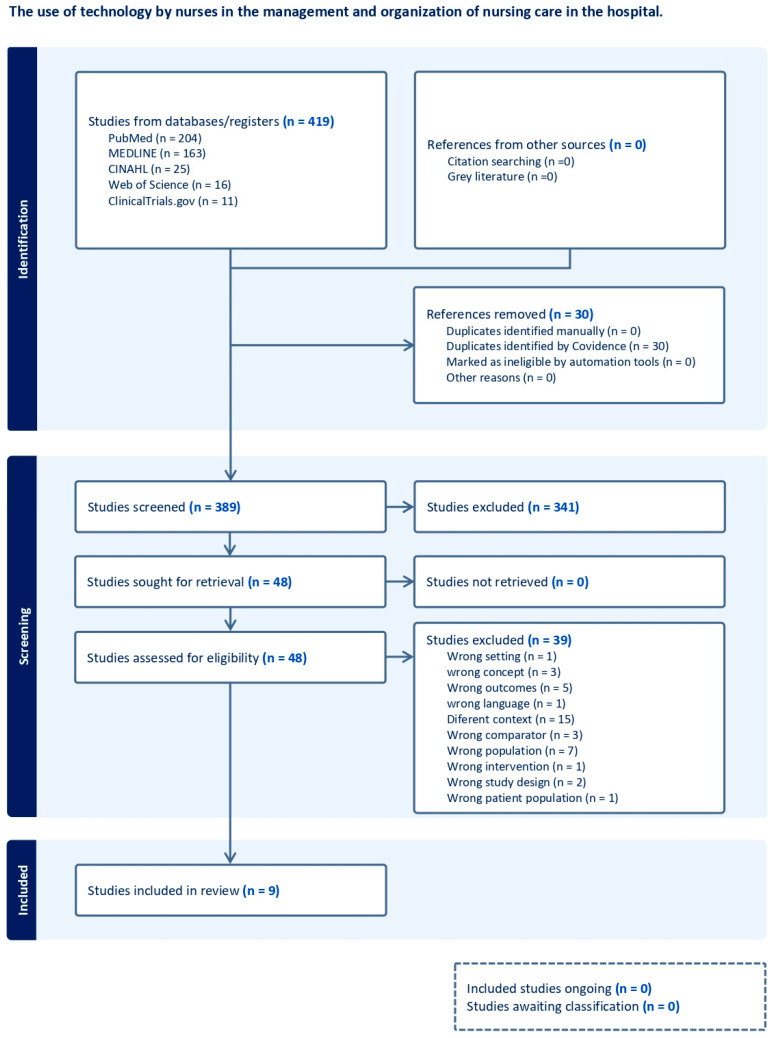
Flowchart of the study selection process.

The results of the search and selection of studies are presented in full (Figure 1), outlined using the Preferred Reporting Items for Systematic Reviews and Meta-analyses ScR (PRISMA-SCR).

At the outset of this process, 419 articles were identified for review analysis, with 30 duplicate articles removed from various databases, leaving 389. After screening titles and abstracts, 48 studies were obtained for full-text review. Finally, nine studies were included in the scoping review, meeting the defined criteria. Of the total excluded articles, thirty-nine did not align with the review objective; sixteen had a different context, three did not correspond to the concept, five had results outside the scope, one did not include the correct language, three compared different contexts, seven did not match the identified population, one was not the intended intervention, one did not correspond to the identified patient population, and two did not correspond to the study design. 

The most frequent conceptual approach was the qualitative approach (*n* = 6), with different methodologies/types of studies: phenomenological (*n* = 1), narrative (*n* = 1), exploratory (*n* = 3), and ethnographic (*n* = 1). This was followed by quantitative (*n* = 2): correlational studies (*n* = 2), exploratory method (*n* = 2), and a review study (*n* = 1). Table 2 summarizes the key information extracted from the studies, including the type of technology used by nurses and its impact on the management and organization of nursing care. 

Nine articles were analyzed in this scoping review, conducted across six countries: Brazil, Canada, China, the United States, England, the Netherlands, and Portugal.

It was identified that hospitals have heavily invested in health information technology, promoted as an integral component of providing quality, safe, and efficient healthcare [23]. According to the Agency for Healthcare Research and Quality, there are more than 5000 types of medical devices in use in healthcare organizations [24], with a strong increase in critical care units. 

### 3.1. Information Technologies Identified

In the field of nursing, the investment in information technology has been notably evident in the computerization of the clinical process, with the development of decision support mechanisms, real-time information availability, integration of individualized indications and prescriptions, clinical alerts, safety equipment for medication dispensing and administration, and the integration of equipment such as monitors, ventilators, infusion pumps, and scanners [25]. 

Patient quality and safety have driven technological evolution, but some articles clearly highlighted the importance of interoperability between information systems in organizations and the integration of national health data registration portals, such as vaccination records [25]. Equipment portability was also identified as an added value in adopting new technologies, particularly because of its impact on nurses’ workflow, significantly influencing nurse–patient interaction, proximity, and patient experience.

### 3.2. Integration of Technology into Organizational Structure and Processes

Redefining organizational policies and procedures is considered a priority, to integrate technological development into the structure and processes of organizations. Reinforcing and giving visibility to the importance of the produced information (data analytics) is suggested to support best practices and, consequently, knowledge development [25,26]. 

It was identified that high-reliability organizations, characterized by a strong safety culture, often facilitate the integration of technology into organizational structure and processes consistently. These are organizations concerned with preventing and evaluating failures, committed to resilience and continuous improvement, as well as legal and privacy issues related to information and access [24]. 

The credibility and influence of system designers with users, system developers, and institutions are identified as essential for their effectiveness in problem solving, mitigating unintended consequences, and positively impacting the safe adoption and use of information technology [27]. The user-centered design principle is considered the foundation for creating usable systems and devices [28]. 

Clinical experience and IT training dedicated to mediating work changes resulting from the implementation of health IT can help mitigate unintended negative consequences [27]. To this end, assigning mediators to ensure that units implementing technology are continually monitored for emerging issues has been identified as highly important [27]. The literature on the sociomateriality of IT is also relevant, with particular attention to literature on working practices, roles, and the visibility of work in organizational power hierarchies [23]. 

The role of leadership was identified several times as crucial in interactions between nurses and other members of the organization. This is to achieve better technology use that improves the patient experience. There is a focus on organizing teams with versatile people, forming mixed teams that include individuals with both more and less experience. This approach allows concerns to be easily communicated and problems to be solved promptly. Technology champions are useful in showing how to use technology, serving as sources of support for teams [29]. Nurse managers can take on this role, being a source of support for the nursing team. Although innovative technologies contribute to management work, it has been described that their effectiveness depends on training, an adequate number of professionals, equipment, and efficient and integrated information systems [30]. 

Team training and support are prioritized across all articles, before, during, and after the implementation process of new technologies. Leadership feedback on user performance is vital for implementation success [26]. Identifying improvements and paying attention to concerns facilitate the acceptability of the system, as well as evaluating and measuring the impact on clinical practice [23,30]. Generally, the teams’ suggestions for improvements are related to the system and equipment.

User acceptance and satisfaction are also of particular importance in the consulted articles [26,29], as well as having a certification program for informatics specialists -nursing informatics [28]. 

Nurses frequently mention organizational factors, such as policies, requirements, leadership decisions, and information for teams, with dissatisfaction. It became evident in some situations that nurses were unaware of policies and the rationale for the absence of some functions in the information systems, such as not allowing the copy/paste function or real-time documentation that expected nurses to record assessments immediately after care. 

### 3.3. How Nurses Use Technology in Care Delivery

Throughout the review, it became evident that the integration of technology into care processes requires significant reconfigurations of work practices at the expense of nurse–patient interaction, leading to the redefinition of priorities regarding care and interaction with patients, with the consequence of delegating articulation work tasks [23]. It was also shown that skills training and team monitoring in technology use has an impact and increase individual and team confidence, reinforcing the importance of training, monitoring, and coaching even after the implementation period of new information systems, pre- and continuous [26,29]. People with more experience in innovation tend to have a more positive attitude toward technology.

Patient experience also drives changes in healthcare due to the accessibility and availability of tools designed for patients today. Nursing must prepare to guide and manage changes in how healthcare and systems process, use, and store patient data. Nurses must be involved at every stage, from redesigning onwards, to ensure the future of technology in patient care [24]. 

Nurses’ behavior arises from tensions in the environment through multidirectional and self-organized interactions. Nurses respond to technological obstacles with self-organized interactions. Self-organization with colleagues is helpful in finding better ways to provide patient care when using technology, with adaptive behaviors to respond to IT obstacles in the workflow. They create workarounds, adopting non-standardized approaches to solving technology-related workflow obstacles, but should not be interpreted as mistakes and errors, detours, or shortcuts [25].

Improvements in processes stand out among the potentialities of using management technologies: ease of record keeping, time management, quick responses, data storage, and patient safety. Technologies have a positive influence on the work of managers, but the problems and difficulties in using them are also significant. It is necessary to understand technologies as work tools used by nurse managers to transform the multiple work objects present in various areas of hospital management, enhance processes, and achieve more effective results [30].

## 4. Discussion

This scoping review shows how the integration of information technology in healthcare organizations impacts management and organizational processes, the care practice environment, nurses’ skills, and patient quality and safety. The organizational structure plays a key role in this area, in terms of reorganizing policies and procedures, communication, investment in resources, team training and support, and identifying strong leaders. Leadership can integrate new information technologies into care processes, with a permanent eye on adapting teams, alignment with nurses’ workflow, and the impact on team performance and satisfaction. Paying attention to team feedback, monitoring, and continuous training, as well as the development of competences, contribute to this. It is also crucial to pay constant attention to the influence of these changes on the quality and safety of care, and on patient’s experience and satisfaction.

Integrating technology into the structure and processes of healthcare organizations at the structural level, the lack of congruence between policies and practice is a source of frustration identified throughout this review, as well as low levels of communication and lack of feedback, which are, as described by Zadvinskis [26], barriers to improving system performance. This result corroborates the importance of system functions not only facilitating the performance of daily clinical tasks, such as quickly obtaining accurate information and data, but also being portable and providing a user-friendly interface, as found in the study by Wang [28]. This goes hand in hand with the need for investment at different levels by the organizational structure, as mentioned in Lalley’s [25] article. There must be explicit policies and procedures that are well communicated to teams, as well as investments in equipment that meets everyone’s needs, as found by Zadvinskis [26]. The application of this is not only for managers to monitor organization activity and assess team satisfaction and performance but also for the user, who, feeling engaged and part of the ongoing process and transformation, contributes to enhancing the organization of care processes. This ensures proximity to the patient and the necessary fluidity for daily operations, so that technology emerges as a support to teams in the continuous pursuit of quality and safety of care, as evidenced by Elgin and Bergero [24].

Regarding management, the importance of strong leadership for organizing processes and teams is highlighted, as found in Berg’s [29] article. The importance of leadership in this process is explicitly stated, referring to team consensus and forming mixed teams, which include people with more and less experience, allowing concerns to be easily communicated and problems to be solved promptly. It is also considered important to identify *technology champions,* to show how to use technology and be sources of support. Nurse managers can take on this role, being a very important source of support for the team and a role model. A nurse manager is responsible for translating the culture and strategy of an organization at the operational level, as well as managing resources, coordinating nursing care, planning and contributing to the evaluation services provided, together with supporting and encouraging teamwork in the relevant units and implementing innovative practices [32]. The competencies of communication, change management, conflict management, motivation, leadership, negotiation and conflict resolution, clinical skills, decision making, strategic thinking, team building strategies, time management, strategic vision, human resources management, information about systems and computers, integrity, legal issues, power and empowerment, professionalism, research and evidence-based practice, and technology were identified as very important requirements for nursing leaders to have the ability to assess the needs of teams and implement appropriate actions for each one [32].

Another aspect mentioned in Vandresen’s [30] article, described as an important step to be taken, was the implementation of macro-policies that enable the teaching–learning process, with the incorporation of technological resources that enhance the training and work processes of future professionals. It becomes clear, therefore, that integrating technology into healthcare organizations goes far beyond the organization itself and the acquisition of innovative technologies. Given the speed at which technological development is occurring today, both at the level of the educational structures that train professionals and in the definition of national and international policies regulating cross-cutting aspects, such as data management and privacy issues, ethical and legal aspects, as identified by Elgin and Bergero [24], the importance of the interconnection between teaching and practice is consolidated so that the knowledge transfer occurs, but guided by policies that ensure the quality and safety of care in healthcare organizations. 

Training, monitoring, and feedback for teams appear in all articles as essential and are considered as having a major impact on the integration of information technology into care processes, with Berg [29] stating that it should be an integral part of the organizational structure when implementing new technologies. This emphasizes the importance of addressing concerns as they arise and providing training. Proceeding in this way will allow the development of skills and the organization of appropriate care processes, rather than self-organized processes and user-created solutions that may compromise the safety of care, as described in the article by Lalley [25], which stated that when nurses encountered obstacles, they created alternative solutions with health information technology. The same article also declares that these are non-standardized approaches to solving a technology-related workflow obstacle, and should not be interpreted as errors or mistakes, deviations, or shortcuts, but they do create tensions in the practice environment.

Also, in Elgin and Bergero’s [24] article, it is described that collaboration and the opinions of the system users are very important for successful implementation. Furthermore, it is shown that patient-centered technological interventions are fundamental for the success of a redesigned healthcare system, which aligns with what is described in the various articles that are part of this review. 

Regarding different information systems, Novak’s [27] article describes that the credibility and efficiency of information technology towards users, system developers, and institutions are essential for its effectiveness in problem solving, mitigating unintended consequences, and positively affecting the adoption and safe use of information technologies. Once again, clinical experience and training in information technologies, when dedicated to mediating work changes resulting from the implementation of health IT, can help mitigate unintended negative consequences. The assignment of mediators to ensure that the units are operational with technology, with continuous monitoring of emerging problems, is described as facilitating and extremely important for the successful implementation of information technology. 

### How Nurses Use Technology to Provide Care

As mentioned in Wang [28] article, the success of the implementation of health information and technology is often linked to the impact the technical system will have on nurses’ work. Throughout several articles in this review, it is described that care processes are often disrupted with the adoption of new technologies [29]. This applies to the use of technology itself, the workflow [26], or the management and organization of care, often requiring reconfigurations of care practice through the adaptation of work practices and technology in the workplace, with real patients [27]. 

While aware that readily available patient assessments and the direct integration of data into the electronic medical record improve work efficiency, as mentioned in Wang [28] article, nurses know that it is necessary to adapt to the complex environment in which they work with innovation, transforming the workflow to meet the needs and goals of patients, as mentioned by Elgin and Bergero [24]. However, this requires a cultural shift because, despite knowing that acquiring information through data will allow us to break down barriers in services, collaborating with others to transition to a paperless practice, it is expected that leadership advocate for system resources to improve nursing workflow so they spend more time with the patient, using strategies to implement new technologies, as described in Zadvinskis et al.’s [26] article. Among other aspects previously identified, it stands out in Vezyridis et al.’s [31] article that strengthening teams during implementation, a reduced patient load, and having individuals more skilled in computer use prove to be a very useful strategic line for adapting to the new system, as well as for unit-specific and interdisciplinary teamwork, corroborated by Zadvinskis [26].

It was also evident in the studies by Lalley [25] and Vezyridis [31] that sharing experiences can help similar organizations with their system implementations. The article by Vandresen [30] on the potential and difficulties mentioned by nurse managers in the use of technology in hospitals shows that the difficulties are related to two elements of the work process: the tools themselves and the workforce. Additionally, there are structural factors and training in the use of technology for efficient management in order to enhance the quality of outcomes.

It has been identified that implementing electronic health records without a focus on usability is the major barrier to their widespread adoption. Experience with technology facilitates end-user acceptance and trust, so it is important to monitor user acceptance levels and supply and provide support when necessary. This involves reorganizing teams with the appropriate resources for different implementation stages, both in terms of the ability to adapt to new technologies, nurses’ knowledge and skills, and in terms of ratios, training, and follow-up. It became clear that adapting work practices and technology in the workplace happens with real patients, and although nurse leaders are aware that nurses are finding ways to adapt health information technology to patient care activities, their focus must be on participating in workflow changes and implementing systems or changes in the IT system. 

**Figure 2:**
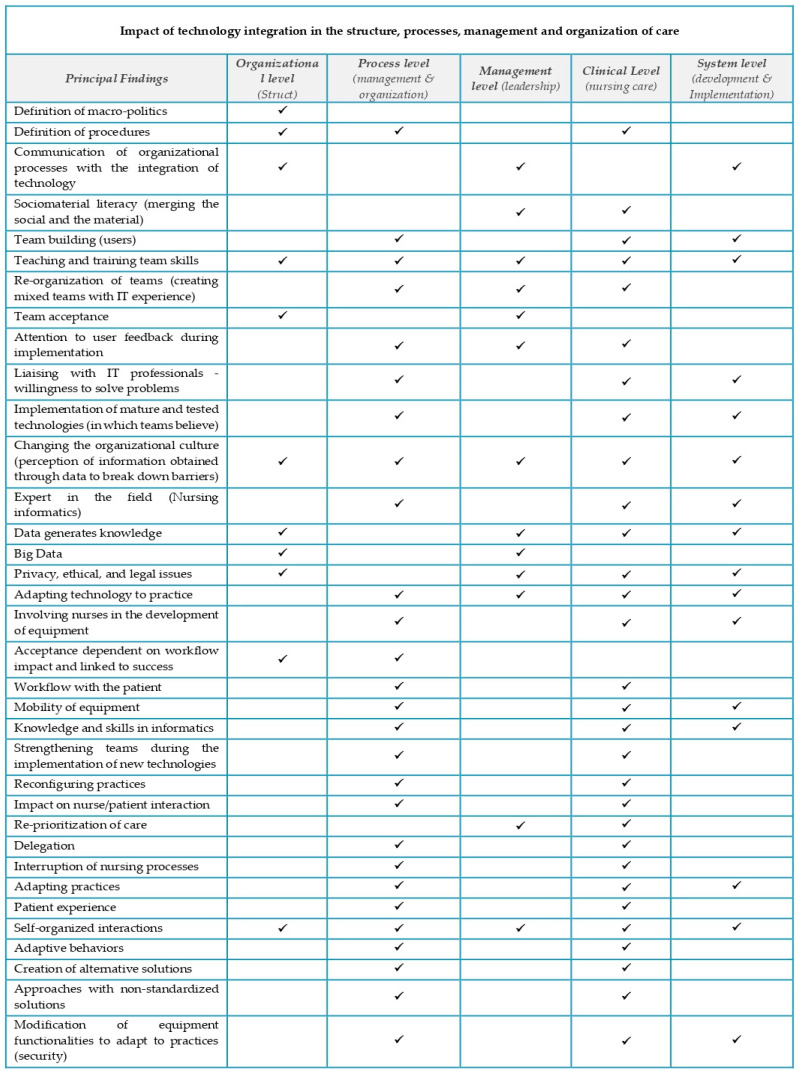
Analysis of the impact of technology integration in the structure, processes, management, and organization of care.

Interactions with patients, caregivers, colleagues, and other elements of the organization are multidirectional and have diverse patterns, reinforcing the importance of organizing the nursing workflow with the patient and solving problems quickly. The introduction of systems that are easy to use, available, and compatible with the processes of the clinical environment, mature enough to allow the team to realize observable benefits immediately, will be facilitative and well received. Improvements in processes stand out among the potentials in using management technologies, in ease of recording, time management, rapid responses, data storage, and patient safety. Technologies positively influence the work of managers, but the problems and difficulties in using them are also significant. With the articles selected for this review, it was possible to identify how the integration of technology in healthcare organizations affects the structure, management, organization of care processes, nursing care, and information systems themselves (Figure 2). 

## 5. Conclusions

It is in the constant fluctuation between stability and instability that organizations change and grow for the better [25]. Therefore, defining the strategy for implementing new technologies requires attention at different levels: at the structural level, defining policies and procedures based on macro-policies, investing in mature systems well accepted by users, a communication plan and selecting leaders; at the process level, organizing processes and teams, identifying the right resources (physical and human), monitoring with a focus on performance, training and skills development, evaluating acceptance; at the results level, practice environment, quality and safety of care, patient experience, and team satisfaction. It was evident throughout this review that, regardless of the information technology adopted, nurses constantly try to adapt in their daily practice to the integration of information technology into care processes, contributing to this with their experience, knowledge, and the skills they develop. However, it was interesting to identify these competences and understand how nurses integrate them into care. Therefore, it is important to carry out another study to identify existing instruments to assess nurses’ technological competences and understand how they are integrated into care processes.

## Figures and Tables

**Table 1:** Data sources and searches.

CINAHL	((Health Information Systems OR Information Systems OR Information Technology) AND (Organization and Administration OR Hospital Administration) AND (Nursing Care OR Nursing OR Patient Care Planning OR Primary Nursing OR Patient Care Management) AND (Nurses OR Nurse Clinicians OR Nurse Administrators OR Nursing, Supervisory) AND (Hospital OR Hospitalization OR Length of stay))
MEDLINE	((Health Information Systems OR Information Systems OR Information Technology) AND (Organization and Administration OR Hospital Administration) AND (Nursing Care OR Nursing OR Patient Care Planning OR Primary Nursing OR Patient Care Management) AND (Nurses OR Nurse Clinicians OR Nurse Administrators OR Nursing, Supervisory) AND (Hospital OR Hospitalization OR Length of stay))
PUBMED	((Health Information Systems OR Information Systems OR Information Technology) AND (Organization and Administration OR Hospital Administration) AND (Nursing Care OR Nursing OR Patient Care Planning OR Primary Nursing OR Patient Care Management) AND (Nurses OR Nurse Clinicians OR Nurse Administrators OR Nursing, Supervisory) AND (Hospital OR Hospitalization OR Length of stay))
SCIELO	((Health Information Systems OR Information Systems OR Information Technology) AND (Organization and Administration OR Hospital Administration) AND (Nursing Care OR Nursing OR Patient Care Planning OR Primary Nursing OR Patient Care Management) AND (Nurses OR Nurse Clinicians OR Nurse Administrators OR Nursing, Supervisory) AND (Hospital OR Hospitalization OR Length of stay))

**Table 2:** Results of individual sources of evidence.

Authors	Type of Study	Population	Type of Technology	Impact on Care Management and Organization
Bergey, 2019 [23]	Qualitative Phenomenological	Nurses and Nurse Managers	Portable device for Electronic Drug Administration Record with CPOE (Computerized Physician Order Entry)	Significant reconfigurations of working practices Re-prioritization of care and interaction with patients. Reorganization of teams with a focus on more versatile people.
Elgin, 2015 [24]	Exploratory and review	Nurses	Electronic medical record Medicine dispensing Infusion pumps ScannersPatient tools	Adaptation of current practices Manage change Involvement in the construction, development, and redesign of systems Safety culture Legal and privacy issues Importance of the information produced Patient-centered technological interventions
Cathy, 2014 [25]	Qualitative with narrative enquiry	Nurses	Electronic medical record Medication barcode dispensing Medication dispensing Vaccine portal	Responding to technological obstacles with self-organized actions Adaptive behaviors to respond to the workflow Create alternative solutions with Information Systems Non-standardized approaches Multidirectional interactions with professional teams and patients Leader facilitates interactions between nurses and other members
Zadvinskis, 2018 [26]	Qualitative longitudinal study	Nurses	Electronic medical records. Barcode medication administration	Investment in equipment Refining policies, procedures, and organizational factors Information/suggestions for/from the teams Leadership feedback on performance Workflow involved in operating systems consistent with internal policies and procedures Workflow with the patient
Novak, 2012 [27]	Qualitative Exploratory with ethnographic method	Nurses	Barcode Medication Administration	Adaptation of working practices and technology in the workplace. Credibility and influence of the systems support team. Clinical experience and training in information systems. Change management with the assignment of mediators.
Wang, 2016 [28]	Quantitative, correlational study	Nurses	Electronic risk assessment system (falls, skin, pain, etc.)	User-centered design Collaboration and the opinions of the system’s users Certification program—nursing informatics as a specialist Evaluate and measure the impact on clinical practice Portable and user-friendly interface improves work efficiency User satisfaction Improving patient safety Data integration
Berg, 2017 [29]	Quantitative, correlational	Nurses	New information technologies in critical care units	Leadership and team consensus Technology champions Mixed teams Team training, skills training, and monitoring Satisfaction and acceptance
Vandresen, 2022 [30]	Qualitative, descriptive–exploratory	Nurse Managers	Information and communication technology (ICT)	Improvements in management processes. Easy registration, time management, quick responses, data storage, and patient safety. Type of technology used by managers. Effectiveness depends on training, an adequate number of professionals, equipment, and efficient, integrated information systems.
Vezyridis, 2012 [31]	Qualitative, exploratory study	Nurses and Nurse Managers	Electronic Health Record	Strategy for implementing new technologies Attention to concerns to facilitate the acceptability of the system. Introduction of user-friendly systems, compatible with the processes of the clinical environment and mature. Sharing experiences of system implementations. System availability and downtime. Technological integration. Transition to a paperless practice. Computer skills.

## Data Availability

The data supporting the results of this study will be available upon request to the corresponding author. The data will not be publicly available due to ethical or privacy restrictions.
